# Implementation of the laboratory strategic framework to strengthen health laboratory services in Eastern Mediterranean Region, 2016–2023

**DOI:** 10.4102/ajlm.v14i1.2611

**Published:** 2025-04-30

**Authors:** Fausta S. Mosha, Rana Hajjeh, Rachel Ochola, Amany Ghoniem, Yvan J.-F. Hutin

**Affiliations:** 1 Department of Communicable Diseases, World Health Organization (Eastern Mediterranean Regional Office), Cairo, Egypt; 2 Director Programme Management, World Health Organization (Eastern Mediterranean Regional Office), Cairo, Egypt; 3 WHO Health Emergencies (WHE) Programme, (Eastern Mediterranean Regional Office), Cairo, Egypt

**Keywords:** laboratory, strategic framework, diagnostic services, health coverage, public health response

## Abstract

**Background:**

Laboratories and diagnostics services are critical to universal health coverage and public health response. We assessed the extent of the implementation and functionality of the 2016–2023 Eastern Mediterranean Region (EMR) laboratory strategic framework.

**Intervention:**

Documents and reports from World Health Organization country offices were examined between September 2022 and November 2022, supplemented by stakeholder-provided documents, to enhance data collection and reporting across the framework’s five goals. An intervention using a performance evaluation scorecard assessed the progress of EMR Member States (MSs) towards strengthening health laboratory services, with findings validated during the December 2022 regional public health laboratory directors’ meeting in Egypt.

**Lessons learnt:**

We analysed results from 21 of 22 MSs. Three (14%) MSs, all high income, had the capacity to implement all indicators, while only one of five low-income (20%) MSs could not demonstrate any capacity across all five goals evaluated. Irrespective of income category, the least implemented domains were: (1) availability of either or both fully implemented laboratory policy, and (2) a fully implemented integrated national laboratory strategic plan, both of which were implemented in only 50% of MSs.

**Recommendations:**

Addressing the identified gaps requires concerted efforts, collaboration, and sustained investment to ensure the delivery of high-quality laboratory services and advance public health outcomes across the EMR. Implementation of laboratory strategies should be coordinated through the specific laboratory department or unit at the Ministry of Health level, above the central public health laboratories and with the support of a national laboratory technical working group.

**What this study adds:**

This study revealed substantial gaps in implementing laboratory policies and strategic plans in the EMR, with full implementation achieved by only 50% of MSs. It underscores the necessity for coordinated efforts and sustained investment to enhance laboratory services and promote effective laboratory practices in EMR.

## Background

Among major global health priorities, three, namely Universal Health Coverage, Antimicrobial Resistance, and the Global Health Security Agenda, require better access to quality laboratory services.^1^ Laboratories establish diagnosis, guide therapy, and monitor disease progression and disease surveillance by offering timely and accurate information.^2,3,4^ Laboratories provide early diagnosis in cases of public health signals, thereby ensuring health security and national development, and fulfilling international obligations, such as those outlined in the International Health Regulations of 2005.^5^ In addition, laboratories contribute to sustainable core capacities and the development of programmes for diseases such as HIV, tuberculosis, and malaria.^6^ There were various initiatives to improve diagnostics in the region, including for 2005–2009,^7^ and for 2010–2011.^8^ Building on previous diagnostic initiatives (2005–2011), the Eastern Mediterranean Region (EMR) focused on improving vaccination coverage in countries with low third dose of the diphtheria, pertussis and tetanus vaccine and district coverage. This included capacity building for healthcare workers and strengthening of immunisation systems. Efforts were also focused on enhanced surveillance for emerging infections and support of high-risk populations. In public health emergencies, technical assistance was provided to meet core epidemiological and laboratory capacities, particularly in areas with high expatriate movement, ensuring timely response and improved disease monitoring.

In 2018, the World Health Organization (WHO) formulated the essential diagnostic list.^9^ However, the pace of advancement has been insufficient. High-income countries have easily adapted their well-organised routine laboratory systems and services, but these are often neglected in intermediate and low-income settings,^10,11,12^ with gaps remaining. Comprehensive national laboratory strategic plans and policies are needed to enhance the laboratory systems, and this is most important in low- and middle-income countries because of the limited resources.^13^ These plans should consider legal and regulatory frameworks, administrative and technical management structure, and effective human resources and retention strategies. Implementation of laboratory quality management systems and the establishment of monitoring and evaluation systems are also required. Prioritisation of procurement and maintenance of equipment, along with infrastructure enhancement, ensure sustainable improvement in national laboratory systems.^14^ Finally, effective leadership, commitment, and coordinated efforts from host governments while fostering public–private partnerships can increase access to quality laboratory services.^15,16,17^

In October 2016, the 63rd session of the Regional Committee for the EMR of the WHO endorsed a strategic framework for strengthening health laboratory services for 2016–2020 through resolution EM/RC63/R.4.^18,19^ The aim was to enhance sustainable national health laboratory systems to improve clinical and public health services, focusing on epidemic-prone diseases, health security, and emergency preparedness. The framework established six strategic goals: strengthening leadership and governance, improving organisation and quality management, ensuring sustainable and competent human resources, ensuring safe and secure laboratory environments, promoting effective laboratory networking, and enhancing coordination and use of laboratory services.

The coronavirus disease 2019 (COVID-19) pandemic profoundly disrupted public health systems, but had mixed effects in terms of laboratory systems strengthening. On one hand, there was unprecedented leapfrogging with concurrent setup of new capacity. In 2020, a surge in polymerase chain reaction testing capacity occurred in response to the urgent need for expanded diagnostic capabilities in the EMR. Subsequently, in 2021, the emergence of viral variants spurred a substantial increase in genomic sequencing capacity to effectively monitor and characterise genetic variations of severe acute respiratory syndrome coronavirus 2.^20^ However, this new capacity failed to include a system to manage the diagnosis and surveillance of other communicable diseases, including antimicrobial resistance. In 2020, the EMR Member States (MSs) temporarily extended the resolution to 2023 to promote a well-defined direction, and to set standards and decision-making on resource (capital and human) allocation. However, the Eastern Mediterranean Regional Office needed a re-assessment before re-designing a new framework. We needed to leverage lessons learnt since 2016, while capitalising on the progress achieved during the COVID-19 response. This article describes overall progress towards the implementation of the EMR Strategic Framework for Strengthening Health Laboratory Services, achievements, challenges, opportunities, and new priorities as the framework resolution came to an end in 2023.

## Description of the intervention

### Ethical considerations

Ethics committee approval was not required for this research. This research involved no human or animal subjects.

### Review of documents

We reviewed available grey literature focused on health system planning, performance and implementation, including documented health policy reports, planning dashboards, performance reports, country implementation plans, periodical reports, and other eligible documentation from the period 2016 to 2022.

### Scorecards

#### Data collected

The WHO Eastern Mediterranean Regional Office developed a performance evaluation indicator-based matrix (or scorecard) to assess the progress made by MSs towards implementation of the Regional Framework for Strengthening Health Laboratory Services. The scorecard focused on the five key goals of the framework (2016 to 2023). We developed specific indicators for the five goals on leadership, legislation and governance, including tiered and integrated laboratory networks (10 indicators). The other indicators were on human resources for laboratory service delivery (8 indicators), and laboratory biosafety and biosecurity (7 indicators). We also developed indicators for the implementation of laboratory quality systems (6 indicators). As the outcome of all five goals will have a direct effect on the rational and evidence-based use of laboratory services, we did not develop indicators for the sixth goal on promotion of rational and evidence-based use of laboratory services. The scores ranged from 1 to 4 (no capacity, limited capacity, developed capacity, demonstrated capacity). Initially, we looked at a high-level overview of the extent of implementation of the priority indicators.

#### Data collection procedure

We sent the scorecards to all 22 WHO country offices in Eastern Mediterranean Regional Office in September 2022, along with instructions on how to fill in the scorecard, with a requested turn-around time of 6 weeks. We also listed the number of documents to be submitted as evidence of the mentioned capacity. The WHO country offices shared the information with the national reference laboratory management, who filled in the scorecard with their quality committee. The director of the national reference laboratory shared the filled-in scorecard together with the attached appendices as evidence of the capacity to the Public Health Laboratory unit in the WHO EMR.

#### Data analysis and validation

We extracted a selection of indicators from each strategic area to plot graphs. The WHO EMR held three laboratory manager’s meetings to validate the results and to plan for the next phase of the strategy to sustainably strengthen the public health laboratory services in the region. The first meeting, held on 05 December 2022 – 07 December 2022,^21^ involved directors of central public health laboratories and partners. Member States reviewed progress and challenges on implementation of the regional framework for strengthening health laboratory services as recorded in the scorecard by the MSs. The second meeting (06 March 2023 – 08 March 2023)^22^ was with technical officers at the central public health laboratories and regional laboratory partners, to deep dive and to agree on the new priorities for the next framework and alternative modalities to strengthen laboratory governance and implementation of laboratory quality management systems. The third meeting (14 August 2023 – 16 August 2023)^23^ was with the policymakers at the Ministries of Health and laboratory regional partners to discuss and deliberate on key policy shifts to attain sustainable delivery of quality laboratory services in the EMR. The deliberation from this meeting provided information to shape the new WHO EMR strategy to strengthen laboratory services.

## Lessons learnt

### Participation in the assessment

We analysed results from 21 of the 22 EMR MSs, representing a range of low (five), lower-middle (seven), upper-middle (three) and high-income (six) economies with varying geographical size and development status. The missing data were from an upper-middle-income country that did not respond before the given deadline.

### Overall scorecard

Laboratory system strengthening varied by MS and by defined goals. Three (14%) MSs, all high-income, had some capacity to implement all indicators, while only one low-income (5%) MS could not demonstrate any capacity across all indicators of the five goals evaluated. Specifically, four (80%) MSs in the low-income group, five (71%) lower-middle income, one (33%) upper-middle income, and one (17%) in the high-income group still had no capacity to implement 19% (6/31) or more indicators. None of the MSs in both low- and lower-middle income brackets had implemented any national staffing plan for their respective networks based on workload forecasting. Implementation of the national laboratory policy and national laboratory strategic plan was least implemented, irrespective of the MS’s income group. Further challenges across the region included underfunding, insufficiently trained laboratory personnel, outdated and poorly maintained infrastructure and equipment, shortages of essential reagents and consumables, inadequate bio-risk management, and limited implementation of quality assurance and quality control measures, among other indicators.

### Leadership and governance

**FIGURE 1:**
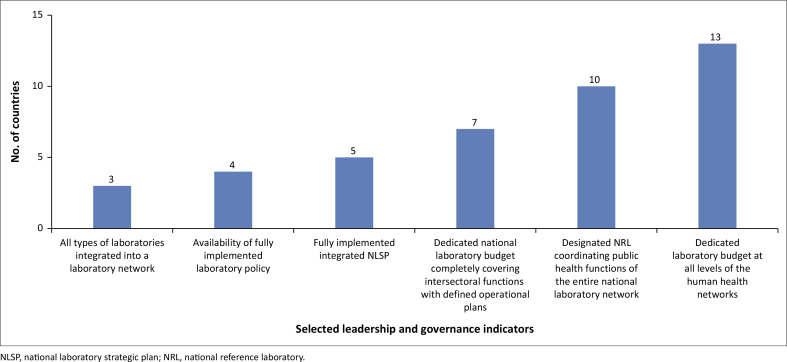
Full implementation of selected leadership and governance indicators, 2022.

The scorecard indicated that only three MSs (14%) have integrated all types of laboratories into a laboratory network, while four MSs (19%) reportedly have a fully implemented laboratory policy in place. It was further noted that five MSs (24%) have successfully implemented an integrated national laboratory strategic plan. Meanwhile, seven MSs (33%) were reported to have a dedicated national laboratory budget that fully addresses intersectoral functions with defined operational plans. Additionally, 10 MSs (48%) were identified as having a designated national reference laboratory responsible for coordinating public health functions across the national laboratory network. Finally, 13 MSs (62%) were reported to have dedicated laboratory budgets at all levels of their human health network (Figure 1).

### Effective, tiered, and integrated laboratory referral networks and enhanced coordination

Six MSs (29%) had a well-established laboratory coordination mechanism at their Ministry of Health, including other multiple ministries and the private sector. Ten MSs (48%) had laboratory networks for all functions with clearly defined tier-specific roles, but only three (14%) integrated all types of laboratories into their networks.

### Human resources for laboratory service delivery

**FIGURE 2:**
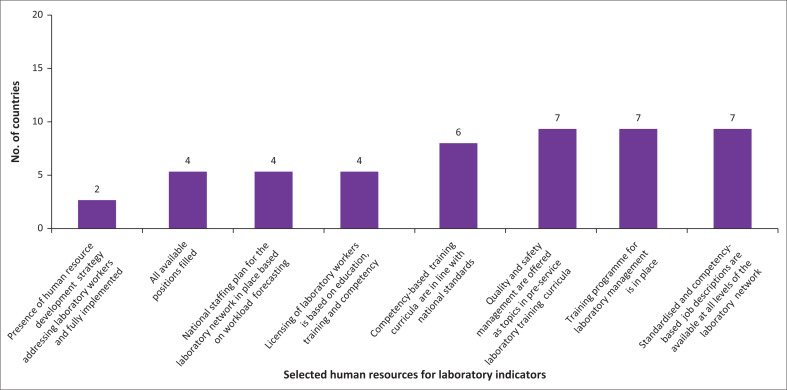
Number of countries fully implemented selected human resources for laboratory indicators, 2022.

Six MSs (29%) had demonstrated capacity with regard to human resources for laboratory service delivery. Additionally, seven (33%) MSs each demonstrated capacity for quality and safety management through various topics in pre-service laboratory training curricula, had pre-service and in-service training programmes for laboratory management, as well as competency-based training curricula in line with national standards. An additional four (19%) had licensed laboratory workers. Licensure was based on education, training and competency assessment, and a national staffing plan for the laboratory network based on workload forecasting (Figure 2).

### Laboratory biosafety and biosecurity

**FIGURE 3:**
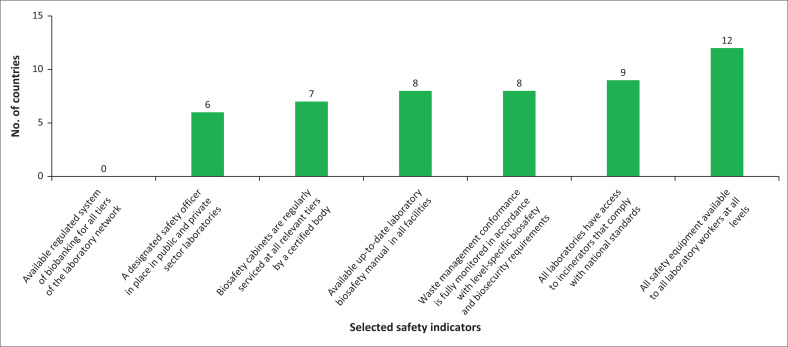
The extent to which selected safety indicators from 2022 have been fully implemented in countries.

Eight MSs (38%) had an up-to-date laboratory biosafety manual in all facilities, while 12 (57%) MSs had all the basic safety equipment available to all laboratory workers and at all levels. Furthermore, six (29%) MSs had appointed safety officers in both public and private sector laboratories, while seven (33%) MSs had certified entities regularly maintaining biosafety cabinets across all relevant tiers. None of the MSs, however, had a regulated system of biobanking implemented at any level of their laboratory systems. Additionally, eight (38%) MSs had established waste management policies in accordance with level-specific biosafety and biosecurity requirements. Finally, all laboratories in nine (43%) of the MSs had access to incinerators that complied with national standards (Figure 3).

### Quality of laboratory services

**FIGURE 4:**
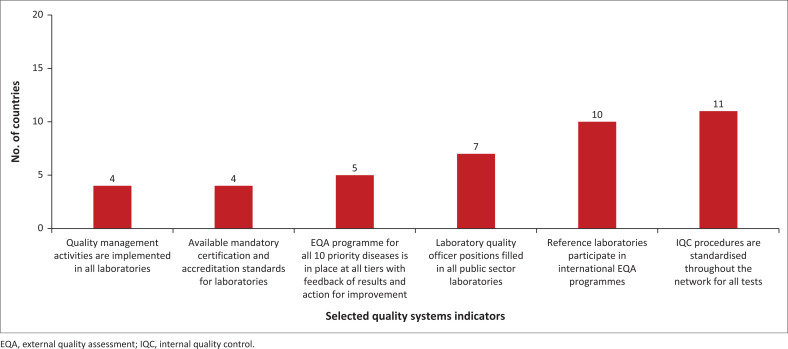
Number of countries that have effectively implemented selected quality systems indicators, 2022.

There were variations on the level of implementation of the laboratory quality management systems (Figure 4). Eleven (52%) MSs had fully implemented standardised procedures for internal quality control across their entire network for all tests carried out. Five (24%) MSs had established an external quality assessment programme for priority diseases across all tiers, facilitating feedback on results and implementing further actions for improvement. Ten MSs (48%) had reference laboratories participating in an international external quality assessment programme, while seven (33%) MSs had filled laboratory quality officer positions in all public sector laboratories. Four (19%) MSs had implemented quality management activities in all laboratories, as well as implementing mandatory certification and accreditation standards for their specific laboratory settings.

## Recommendations

Results of this review point to the multifaceted landscape of laboratory system strengthening efforts and capacity levels across MSs in the EMR towards defined goals. Few MSs exhibited robust capacity on the implementation of the framework. However, we identified limited laboratory capacity in some MSs that represents a barrier to implementation and sustainable diagnostic services. The gaps persisted, particularly concerning the implementation of national strategic planning frameworks.

Leadership and governance structures were heterogeneous among MSs, pointing to variations in investment levels in laboratory budgets and stages of policy implementation. Despite the availability of dedicated laboratory budgets in over 50% of the MSs, and designated national reference laboratories, 76% (16) MSs had yet to fully actualise developed policies and strategic plans. To realise the full implementation of related indicators that are still lagging, these still need to trickle down to all levels of leadership and governance. These challenges may be exacerbated by the lack of national-level monitoring of laboratory service performance. Furthermore, laboratories often do not receive sufficient priority and recognition within national health systems. Organisational bottlenecks, including insufficient leadership and governance, can influence the health system.^24,25^ In the absence of national laboratory strategies, it is difficult to provide comprehensive national laboratory services.

The importance of laboratories was appreciated during the COVID-19 pandemic, which exemplified the need for timely and quality laboratory services. In response to COVID-19’s urgent laboratory needs, investments by governments and partners have strengthened laboratory services and systems in many countries. However, these have often been focused, mainly intended to address COVID-19.^26^ To strengthen and sustain laboratory services, laboratory services must be integrated at various levels of the laboratory network. The evidence-based integrated and tiered laboratory network would form the basis for improved laboratory services.^1^ Several MSs showcased well-established coordination mechanisms and tiered networks. However, there are still existing challenges with integrating laboratory services. The implementation of a network approach has provided greater cost savings, efficiencies, increased coverage, and increased access,^27^ and hence improved network functionality and coordination mechanisms. A comprehensive array of human resources for health directed towards the promotion or improvement of human health is needed to achieve universal health coverage by 2030.^28^ However, in the EMR, human resources capacities for laboratory service delivery presented a complex landscape, with fewer than half of the MSs demonstrating proficiency. While there were commendable efforts in incorporating quality and safety management topics in training curricula, and in providing pre-service and in-service training programmes, challenges persisted in developing and implementing national staffing plans and competency-based training curricula.

Laboratory biosafety and biosecurity measures presented another area of concern, despite notable progress on the provision of personal protective equipment for staff. Strides have been made in safety equipment availability and the appointment of safety officers, but critical deficiencies remained, including the absence of regulated biobanking systems and the imperative for enhanced waste management policies aligned with biosafety and biosecurity requirements. To ensure the safety of personnel and the environment, MSs will need to develop and implement a national regulatory framework that addresses biosafety and biosecurity with the inclusion of other sectors, such as animal health and environment.

Overall, our analysis underscores the complex nature of laboratory system strengthening efforts within the EMR, characterised by an array of achievements and challenges. Addressing the identified gaps demands concerted efforts, collaboration, and sustained investment to ensure the delivery of high-quality laboratory services and advance public health outcomes across the EMR. To sustain the gains from 2016 to 2023, with substantial investments during the COVID-19 pandemic, MSs will need to support laboratory systems as a priority, by developing a national laboratory policy within the national health development plan that will guide the implementation of a national integrated laboratory strategic plan. The implementation of laboratory strategy will be coordinated through the specific laboratory department or unit at the Ministry of Health level, above the central public health laboratories. This will be able to oversee both public health and clinical laboratory services. This can go hand-in-hand with the creation of the multisectoral and multi-programme national laboratory technical working group to better coordinate and integrate laboratory services in the MSs. The new laboratory framework to strengthen laboratory services in the EMR (2024–2029) therefore prioritises laboratory leadership and governance, laboratory quality management systems, and biosafety and biosecurity. The WHO EMR will coordinate the efforts of MSs through the establishment and technical support of a regional laboratory technical working group with representation from MSs and partners.
